# More than a skin disease: stress, depression, anxiety levels, and serum neurotrophins in lichen simplex chronicus^☆^^☆☆^

**DOI:** 10.1016/j.abd.2021.04.011

**Published:** 2021-10-04

**Authors:** İlknur Kıvanç Altunay, Ezgi Özkur, Ece Uğurer, Ecem Baltan, Çiğdem Aydın, Erdinç Serin

**Affiliations:** a Department of Dermatology, Şişli Etfal Training and Research Hospital, Health Sciences University, Istanbul, Turkey; b Department of Biochemistry, Şişli Etfal Training and Research Hospital, Health Sciences University, Istanbul, Turkey; c Department of Psychodermatology, Şişli Etfal Training and Research Hospital, Health Sciences University, Istanbul, Turkey

**Keywords:** Anxiety, Depression, Neurodermatitis, Psychodermatology

## Abstract

**Background:**

Lichen simplex chronicus is a dermatological condition due to excessive scratching, with few studies on psychoneuroimmunology.

**Objective:**

We aimed to estimate the levels of stress, depression, and anxiety, and to measure serum levels of neurotrophins in patients with lichen simplex chronicus, and to correlate these parameters with the severity of the disease and pruritus.

**Methods:**

Thirty-six patients with lichen simplex chronicus and 36 age- and sex-matched healthy controls were included. Each participant was administered the Hospital Anxiety and Depression Scale and Perceived Stress Scale questionnaires, along with a visual analog scale for pruritus. Levels of neurotrophins (brain-derived neurotrophic factor, neurotrophin-3, nerve growth factor, glial cell line-derived neurotrophic factor) were determined by ELISA assays.

**Results:**

The scores of Perceived Stress Scale-10, Hospital Anxiety and Depression Scale were statistically higher in patients (p < 0.05 for all). The serum levels of all neurotrophins were significantly lower in patients compared to healthy controls (p < 0.05 for all). Disease severity showed no correlation with all four neurotrophins. In linear regression models applied for increased visual analog scale-pruritus scores and disease severity these two variables were statistically significant predictors (p = 0.043).

**Study limitations:**

A direct causal relationship was not addressed.

**Conclusion:**

Lichen simplex chronicus patients are at risk of increased levels of stress, anxiety, depression, and present decreased levels of neurotrophins, that may suggest a role in the pathophysiology of this disorder.

## Introduction

Lichen Simplex Chronicus (LSC), or circumscribed neurodermatitis, is a common dermatological disorder characterized by lichenification of the skin due to excessive scratching and/or rubbing caused by the itch-scratch-itch cycle, which is hard to break. It has been estimated that LSC affects up to 12% of the total population.^1^ Although some authors believe that it is a chronic and localized form of atopic dermatitis, its pathogenesis is still unclear. Previous reports indicate that psychological factors may play a role in the initiation of the disease and its persistence. Patients with LSC have proportionally higher incidences of depressive, dissociative, and anxiety disorders, obsessive-compulsive personality traits, sleep disturbances, and sexual dysfunction.^2^, ^3^, ^4^, ^5^ An increased likelihood of comorbid mental health disorders, including developmental, psychotic, and mood disorders, substance abuse, and cognitive disorders were reported in LSC.^6^

Skin is the largest, most innervated organ of the body, and the brain shares a common embryological origin with the ectoderm. The central nervous system (CNS) responds to psychological stress by activating the Hypothalamic-Pituitary-Adrenal axis (HPA axis) and autonomic nervous system and by modulating microglia at a local level. Neurotrophins, such as Brain-Derived Neurotrophic Factor (BDNF), Neurotrophin-3 (NT-3), Nerve Growth Factor (NGF), and Glial cell line-derived Neurotrophic Factor (GDNF), show neuroprotective effects in the CNS and play a crucial role in the growth, differentiation, maintenance, and synaptic plasticity of the neuronal and immune systems. Abnormalities in the levels of these neurotrophic factors may contribute to the dysfunction of astrocytes and microglia.^7^ This may, in turn, directly create some alterations in the skin or induce the release of immunological or hormonal mediators that cause pathological changes in the skin by upsetting the balance of nervous system mediators.^8^ Several studies have confirmed increased levels of NGF or BDNF in atopic dermatitis, chronic spontaneous urticaria, symptomatic dermographism, acne vulgaris patients with depressive symptoms, and uremic patients with pruritus.^9^, ^10^, ^11^, ^12^, ^13^, ^14^

The aim of the present study was to investigate depression, anxiety, quality of life, and perceived stress levels, and to measure serum neurotrophin levels in patients with LSC, and to determine whether there is a relationship between these parameters and the severity of symptoms.

## Methods

This cross-sectional, case-control study was carried out between October 2019 and November 2020. Thirty-six patients diagnosed with LSC and 36 healthy age- and sex-matched subjects were included in the study. Individuals under 18 years old, with any other skin/systemic disease or mental disorder, or on any medication were excluded from the study. The study was approved by the ethics committee (n° 1347). All participants were given informed consent prior to participation and examined by the same dermatologists. Relevant investigations and/or skin biopsies were performed to confirm the diagnosis of LSC when indicated. The severity of the disease (mild, moderate, or severe) was recorded.^15^ The demographics, severity of the disease, and results of the Dermatology Life Quality Index (DLQI), HADS,^16^ PSS-10, and VAS-Pruritus were recorded for each participant.^16^, ^17^ Serum IgE levels were determined using an automated chemiluminescence assay; NGF, NT-3, GDNF, and BDNF levels were determined using ELISA kits. Full details of the questionnaires, ELISA, and statistical analyses are attached in Supplementary materials.

## Results

The demographics and disease characteristics of the study group are shown in Table 1. The mean age was 37.8 ± 11.9, and the most common localization was the back (n = 16. 44%). The PSS-10, HADS-A, and HADS-D scores were statistically higher in the LSC group. The laboratory parameters of all neurotrophins were significantly lower in the LSC group (Table 2). The mean values of serum IgE and the peripheral blood eosinophil count did not differ between groups. A correlation analysis showed that disease severity (*r* = 0.366, p = 0.028) and HADS-D score (*r* = 0.378, p = 0.023) correlated with the VAS-Pruritus score, whereas the HADS-A score correlated with disease severity (*r* = 0.358, p = 0.032). Disease severity showed no correlation with the serum levels of all four neurotrophins (p > 0.05 for all) (Table 3). Linear regression analyses to assess the contribution of the scores of all implemented questionnaires (HADS, PSS-10, DLQI) and the serum levels of neurotrophins as well as serum IgE and eosinophil counts to VAS-Pruritus scores and disease severity are shown in Table 4, Table 5. Disease severity was statistically associated with the VAS-Pruritus score ( p = 0.043 and β = 0.396).

**Table 1:** Demographic and clinical characteristics of the Lichen simplex chronicus (LSC) and control groups.

		LSC	Controls	p
		(n = 36)	(n = 36)	
**Sex, n (%)**	Male	7 (19.4)	7 (19.4)	1.000
	Female	29 (80.6)	29 (80.6)	
**Age (yrs) Mean ± SD (Min–Max)**		37.8 ± 11.9 (19–59)	37.8 ± 11.9 (19–59)	1.000
**Marital status, n (%)**	Single	15 (41.7)	13 (36.1)	.635
	Married/cohabitating	20 (55.6)	23 (63.9)	
	Divorced/widowed/separated	1 (2.8)	0 (0.0)	
**Disease duration (yrs.) Median (IQR)**		3 (1–7)		
**VAS-Pruritus Median (IQR)**		7 (6–9)		
**DLQI Median (IQR)**		4.5 (3–13)		
**Disease severity, n (%)**	Mild	10 (27.7)		
	Moderate	14 (38.8)		
	Severe	12 (33.3)		
**Localization, n (%)**	Head	4 (11.1)		
	Front chest	4 (11.1)		
	Back	16 (44.4)		
	Upper extremities	5 (13.9)		
	Lower extremities	13 (36.1)		
	Gluteus	8 (22.2)

**Table 2:** Comparison of questionnaire scores and laboratory parameters between Lichen simplex chronicus (LSC) and control groups.

	LSC	Controls	p
	Mean ± SD (Min-Max)	Mean ± SD (Min-Max)	
**PSS-10**	18.3 ± 6.4 (0–28)	12.6 ± 5.1 (2–23)	< 0.001
**HADS-A**	8.28 ± 4.65 (1–19)	5.28 ± 2.94 (0–10)	0.002
**HADS-D**	7.39 ± 4.93 (0–20)	4.17 ± 2.48 (0–13)	0.001

	Median (IQR)	Median (IQR)	
**IgE (mg/dL)**	36.7 (8.3–121)	58.1 (21.2–100)	0.374
**Eosinophils (×106/mL)**	0.13 (0.06–0.27)	0.12 (0.08–0.18)	0.969
**NGF (ng/mL)**	141.7 (80.3–689.9)	437 (148.4–1,142.0)	0.010
**GDNF (ng/mL)**	1.61 (0.88–11.63)	5.24 (1.80–20.26)	0.019
**NT (pmoL/L)**	49 (27.1–143.1)	98.2 (47.2–242)	0.012
**BDNF (ng/mL)**	0.88 (0.71–1.87)	1.34 (0.92–2.43)	0.004

**Table 3:** Spearman’s correlation between VAS, disease severity, and study parameters.

	VAS-Pruritus		Disease Severity	
	r	p	r	p
**Disease severity**	0.366	0.028		
**Age (yrs)**	-0.088	0.610	-0.031	0.857
**Disease duration (yrs)**	-0.019	0.912	0.266	0.116
**DLQI**	0.319	0.058	0.359	0.031
**PSS-10**	0.231	0.175	0.218	0.201
**HADS-A**	0.309	0.067	0.358	0.032
**HADS-D**	0.378	0.023	0.107	0.536
**IgE (mg/dL)**	0.203	0.234	0.118	0.494
**Eosinophils (×106/mL)**	0.132	0.442	0.069	0.689
**NGF (ng/mL)**	-0.055	0.748	-0.103	0.548
**GDNF (ng/mL)**	-0.055	0.750	-0.135	0.433
**NT (pmoL/L)**	-0.020	0.906	-0.073	0.673
**BDNF (ng/mL)**	0.016	0.925	-.030	0.863

**Table 4:** Linear regression model for variables identified as significant for increased VAS-Pruritus score.

Model	Unstandardized Coefficients B	Standardized Coefficients β	p
Constant	2.978		
**Disease severity**	0.376	0.396	0.043
Sex	-1.154	-0.206	0.290
Age	-0.027	-0.144	0.481
DLQI	0.061	0.151	0.395
PSS-10	-0.019	-0.055	0.841
HADS-A	0.088	0.182	0.444
HADS-D	0.168	0.369	0.133
IgE	-0.001	-0.246	0.146
Eosinophils	1.596	0.110	0.550
NGF	0.000	0.131	0.769
GDNF	0.032	0.310	0.340
NT	0.002	0.356	0.319
BDNF	1.634	0.688	0.393

**Table 5:** Linear regression model for variables identified as significant for disease severity.

Model 1	Unstandardized Coefficients B	Standardized Coefficients β	p
Constant	0.970		
**VAS**	0.480	0.455	0.043
Sex	0.820	0.139	0.509
Age	0.007	0.036	0.870
DLQI	0.059	0.138	0.470
PSS-10	0.044	0.118	0.685
HADS-A	0.094	0.184	0.471
HADS-D	-0.205	-0.427	0.103
IgE	0.002	0.302	0.093
Eosinophils	-1.151	-0.076	0.703
NGF	-0.002	-0.420	0.377
GDNF	-0.027	-0.251	0.472
NT	0.000	0.053	0.891
BDNF	1.144	0.457	0.599

## Discussion

Our results showed increased stress, anxiety, and depression in LSC group compared to controls. Perceived stress refers to the feelings or thoughts that an individual has about how much stress they are under at a given point in time or over a given period. Theoretical definitions of stress highlight the role of perception as a crucial factor in its experience.^18^ The PSS measures individuals’ perception of stress over the past month. The fact that patients with LSC had higher PSS scores than the controls support the idea that LSC patients have higher mental distress and thus that mental stress may play a role in LSC. Chronic stressors can promote psychoneuroimmunological changes via the HPA axis, resulting in an exacerbation of current skin conditions or the development of new ones. These conditions may impact one’s quality of life negatively, resulting in a decline in psychological wellbeing and an exacerbation of current psychiatric disorders or the formation of new ones. Additionally, high VAS-Pruritus scores (mean=7, min-max 6–9) were shown in LSC, that were correlated with disease severity and HADS-D scores. Therefore, the severity of itch, disease and depression played a role in the LSC process. Dalgard et al. reported that the presence of itch in dermatological patients was significantly associated with clinical depression, suicidal ideation, and stress in diverse countries.^19^ Yamamato et al. investigated the link between pruritic symptoms and psychological stress using PSS, and a relationship between the severity of pruritus and psychological symptoms was shown in the general population.^20^ Yalçın et al. found that 62% of 50 LSC patients had at least one psychiatric comorbidity, mostly major depressive disorder (32%), dysthymia (18%), and generalized anxiety disorder (12%).^21^ Halvorsen et al. stated that itch is a greater risk factor for psychological problems compared to chronic eczema without itch.^22^

All these findings beg the question of whether the psychoneuroimmunological system plays any pathogenetic role in the relationship between itch and psychological stress and thus between LSC and psychological stress. After all, the neurological, endocrine and immune systems interact with each other to develop a sufficient stress response for the body and mind by utilizing a series of neurotrophins, neuropeptides, neurotransmitters, and neurohormones.

Neurotrophins belong to a family of trophic factors that regulate the survival, growth, and programmed cell death of neurons.^23^ They exert immunomodulatory functions not only on nerves but also on immune cells, which was previously proven for NGF.^24^ Some neurotrophins were studied in different dermatological conditions.^9^, ^10^, ^12^, ^13^, ^14^ In several skin chronic inflammations, such as atopic dermatitis, psoriasis, and prurigo, NGF expression was increased in the lesions.^25^, ^26^, ^27^ In fact, the number of peripheral nerve fibers and the concentration of NGF in LSC lesions have been reported to be higher. In the present study, it is noteworthy that all neurotrophins, including NT-3, NGF, GDNF, and BDNF, were significantly lower in LSC patients compared to healthy controls. This may mean that these declines were far from chance occurrences. Furthermore, neurotrophins, especially BDNF and NT-3, have been investigated in mood disorders and found to play a role in depression and anxiety.^28^ He et al. showed that serum BDNF levels were lower and negatively associated with depressive symptoms in young Chinese adults with acne vulgaris.^13^ They also found that the severity of the disease was not related to depression scores in their acne patients, as did we in LSC. In the present study, all neurotrophins were no correlated with LSC severity.

Finally, LSC patients had increased levels of perceived stress, anxiety, and depression, and also decreased levels of neurotrophins, which play a significant role in brain and nerve chemistry. However, the question of which came first, LSC or these psychoneuroimmunological issues were not addressed. Decreased neurotrophin levels might lead to depression or anxiety and may trigger LSC, or conversely, LSC might cause depression or anxiety by reducing the quality of life, which may in turn decrease neurotrophins. It might even be a two-way relationship. These results may be used to help understand the disease better, shine a light on its pathogenesis, or discover new therapy options for LSC in the future.

## Conclusion

The present study showed that LSC patients are at risk of increased levels of stress, anxiety, depression, impaired quality of life and decreased levels of neurotrophins. Decreased serum levels of neurotrophins may be directly related to the etiology of LSC or associated with the high percentage of psychiatric disorders in that patient population. Future studies are needed to address the psychoneuroimmunological basis of LSC and for new therapies.

## Financial support

Health Sciences University, coordinatorship of scientific research projects.

## Authors’ contributions

İlknur Kıvanç Altunay: Approval of the final version of the manuscript; manuscript critical review; study conception and planning.

Ezgi Özkur: Critical literature review; preparation and writing of the manuscript; statistical analysis.

Ece Uğurer: Data collection; analysis and interpretation.

Ecem Baltan: Data collection; analysis and interpretation.

Çiğdem Aydın: Effective participation in research orientation.

Erdinç Serin: Manuscript critical review.

## Conflicts of interest

None declared.

## Appendix A Supplementary data

Supplementary data to this article:
