# Supplementary material for: More than a skin disease: stress, depression, anxiety levels, and serum neurotrophins in lichen simplex chronicus
Source: An Bras Dermatol. 2021 Oct 4;96(6):700–5. doi: 10.1016/j.abd.2021.04.011 (PMC8790192; doi:10.1016/j.abd.2021.04.011)
Supplement: Supplementary file 1 [file mmc1.docx]

***Questionnaries***

Each participant was asked to complete a two part document. The first part contained questions about socio-demographic variables: sex, age, marital status, disease duration; the second part contained the following questionnaires:

**The Dermatologic Life Quality Index (DLQI):** Consisting of ten questions about symptoms, embarrassment, shopping and home care, clothes, social and leisure, sport, work or study, close relationships, sex and treatment over the last week. Each question is scored from 0 to 3, giving a possible score range from 0 (no impact of skin disease on quality of life) to 30 (maximum impact on quality of life). Categories are: 0-1: no effect at all on patient’s life, 2-5: small effect on patient’s life, 6-10: moderate effect on patient’s life, 11-20: very large effect on patient’s life, 21-30: extremely large effect on patient’s life.

**The Hospital Anxiety and Depression Scale (HADS);** is a well-validated scale to assess depression and anxiety. It includes seven items assessing anxiety and seven for depression, each with four possible responses. For each dimension of anxiety and depression, a score from 0 to 7 is considered a normal case, from 8 to 10 a borderline case, and from 11 to 21 a case in need of further examination or requiring treatment.

**The Percieved Stress Scale (PSS-10)** measures important components of stress by assessing how uncontrollable, overloaded, and unpredictable individuals find their lives. The PSS-10 asks about thoughts and feelings over the last month using a response scale from 0 (never) to 4 (very often).

**Visual Analogue Scale (VAS)- Pruritus:** This scale provides a non validated but simple descriptive profile of pruritus for participants’ self-rated health on a vertical visual analogue scale from 0 to 10 (0 = able to cope with normal activities, 10 = completely incapacitated due to pruritus).

***Labaratory Tests***

Study subjects were accommodated comfortably in a climate controlled room with a temperature maintained at approximately 23 °C in the morning (before 9.30 AM). All participants were non-fasting in during the sampling. Then, the blood sample coagulated at room temperature for 10-20 minutes and centrifuged at 2000-3000 rpm for 20 minutes at 4°C. All serum samples were collected and then stored for less than 6 months at − 80 °C. Sample concentrations in each plate were calculated from standard curves and dilution factors. Following factors were studied.

**BDNF:** The measurement was performed by ELISA according to the manufacturer’s instructions (YL Biotech Co., Shanghai, China), and microplate reader BIO-TEK ELX50, (Vermont, ABD) were used to determine BDNF values. Mean intra-assay and inter-assay coefficients of variation were 0.05-10 ng/ml respectively. The assay sensitivity threshold was 0.01 ng/ml.

**NGF:** The measurement was performed by ELISA according to the manufacturer’s instructions (YL Biotech Co., Shanghai, China), and microplate reader BIO-TEK ELX50, (Vermont, ABD) were used to determine BDNF values. Mean intra-assay and inter-assay coefficients of variation were 0.05-20ng/ml respectively. The assay sensitivity threshold was 0.02 ng/ml.

**GDNF:** The measurement was performed by ELISA according to the manufacturer’s instructions (YL Biotech Co., Shanghai, China), and microplate reader BIO-TEK ELX50, (Vermont, ABD) were used to determine BDNF values. Mean intra-assay and inter-assay coefficients of variation were 0.05-20ng/ml respectively. The assay sensitivity threshold was 0.02 ng/ml.

**NT-3:** The measurement was performed by ELISA according to the manufacturer’s instructions (YL Biotech Co., Shanghai, China), and microplate reader BIO-TEK ELX50, (Vermont, ABD) were used to determine BDNF values. Mean intra-assay and inter-assay coefficients of variation were 2-600pmol/l sensitivitesi respectively. The assay sensitivity threshold was 1.04pmol/l.

**IG-E:** The measurement was performed by chemiluminescent immunometric assay (IMMULITE 2000 Total IgE). The mean reference range was 20.4 (IU/mL).

***Statistical Analysis***

Data were analyzed using IBM SPSS 15.0 for Windows v.21.0. (IBM Corp., Armonk, NY). Descriptive statistics are given as number and percentage for categorical variables, and average and standard deviation for numeric variables when appropriate. When parametric assumptions were met, the independent Student’s t test was used to compare numeric variables between patients and controls, and the Mann–Whitney U test was used to compare numeric variables when parametric assumptions were not met. The Chi-square test was used to compare between group differences in categorical variables. Spearman’s correlation coefficient was used to analyze the association between numerical variables. Lineer regression analysis was used for determinants. Linear regression were expressed as regression coefficient (beta) and 95% confidence interval (95%CI). The statistical alpha (level of significance) level was accepted as p<0.05.
